# Feature selection for fMRI-based deception detection

**DOI:** 10.1186/1471-2105-10-S9-S15

**Published:** 2009-09-17

**Authors:** Bo Jin, Alvin Strasburger, Steven J Laken, F Andrew Kozel, Kevin A Johnson, Mark S George, Xinghua Lu

**Affiliations:** 1 Medical University of South Carolina, Charleston, SC, USA; 2 Cephos Corporation, Pepperell, MA, USA; 3 University of Texas Southwestern Medical Center, Dallas, TX, USA

## Abstract

**Background:**

Functional magnetic resonance imaging (fMRI) is a technology used to detect brain activity. Patterns of brain activation have been utilized as biomarkers for various neuropsychiatric applications. Detecting deception based on the pattern of brain activation characterized with fMRI is getting attention – with machine learning algorithms being applied to this field in recent years. The high dimensionality of fMRI data makes it a difficult task to directly utilize the original data as input for classification algorithms in detecting deception. In this paper, we investigated the procedures of feature selection to enhance fMRI-based deception detection.

**Results:**

We used the t-statistic map derived from the statistical parametric mapping analysis of fMRI signals to construct features that reflect brain activation patterns. We subsequently investigated various feature selection methods including an ensemble method to identify discriminative features to detect deception. Using 124 features selected from a set of 65,166 original features as inputs for a support vector machine classifier, our results indicate that feature selection significantly enhanced the classification accuracy of the support vector machine in comparison to the models trained using all features and dimension reduction based models. Furthermore, the selected features are shown to form anatomic clusters within brain regions, which supports the hypothesis that specific brain regions may play a role during deception processes.

**Conclusion:**

Feature selection not only enhances classification accuracy in fMRI-based deception detection but also provides support for the biological hypothesis that brain activities in certain regions of the brain are important for discrimination of deception.

## Background

Blood oxygen level dependent (BOLD) fMRI [1] has been widely used to detect brain activity. Recently, brain activation patterns detected by fMRI technology have been used as biomarkers to detect deception (lie detection) [2-10]. Currently, the existing rule-based classification methods for deception detection have already achieved excellent performance [5,6,9,10]. In these studies, regions of interest were first identified through performing conventional group-wise statistical comparisons of brain activation patterns obtained during lying and truth-telling sessions. Then, the activation patterns within the regions were used as input features to derive various classification rules. Machine learning algorithms have also been applied to perform deception detection based on fMRI data [8]. The task is commonly cast as a classification problem, in which pre-processed BOLD signals in the voxels of fMRI images are treated as input features and each input image is associated with a class label. Using fMRI image data as input for classification poses a major challenge due to the high dimensionality of fMRI images. The images usually consist of hundreds of thousands of voxels that causes most of the contemporary classification algorithms to suffer from overfitting – a phenomenon whereby a classifier performs well on training data but fails on new data. In this study, we investigated the utility of different dimension reduction and feature selection procedures in fMRI-based deception detection. While this study concentrates on employing fMRI-based biomarkers for deception detection, the principle developed in this study can be applied to other fMRI-based biomarker for translational research, e.g., psychiatric disease diagnosis and prognosis.

## Results and discussion

### Classification without feature selection

When dealing with high dimensional data, two types of methods can be applied: dimensional reduction and feature selection. We first investigated a few models that perform high dimensional classification without selecting features. Contemporary state-of-the-art classifiers, particularly partial least square (PLS) [11], random forest (RF) [12] and support vector machine (SVM), have achieved excellent performance in various high-dimensional classification tasks, e.g., text categorization [13] and microarray-based disease classification [14-16]. The PLS algorithm deals with high dimensional data by reducing the dimensionality of the data through a linear project of data from a high dimensional space to a low dimensional principal component space, with a constraint of maintaining the class separation in the low dimensional space. RF handles high dimensionality by averaging the output from a large number of classification trees trained with relatively small number of features. SVM employs kernel methods to project training data into a high dimensional space to make the data points separable and reduces overfitting through maximizing the margin that separates the data from different classes. Since each classification task is unique, we first investigated how well these classifiers handled the high dimensional data and if they performed well over the fMRI-based deception detection. The PLS and RF classifiers ran extremely slowly on the data with 65,166 features, which would render them impractical in the real world application. We then tested the methods on the data consisting of 1,070 voxels selected in our previous study [5] as features. We applied leave-one-out cross validation to estimate the accuracy, sensitivity and specificity. Briefly, the performances of the above classifiers were not satisfactory in terms of overall accuracy. In PLS classification, models with different number of principal components, from 5 to 10 and 15, were trained. All models showed accuracy less than 60%. In the case of the RF classifier, models were trained with 2,000 trees and the number of features for each tree varied from 5 to 10, 50, and 100. The accuracies for all trained models were smaller than 60%, which were deemed to be non-satisfactory. Lastly, the accuracy of SVM with the leave-one-out evaluation was 55.2%. The above results indicate that the fMRI-based classification is a unique task, in which directly applying conventional classification algorithms in an out of box manner does not perform well. The results also indicate that dimension reduction using PLS and RF failed to provide practically acceptable performances.

Another approach to reduce the dimensionality of the fMRI data is to sample the BOLD signals using a large voxel size. In a recent report on fMRI-based deception detection by Davatzikos *et al *[8], the dimensionality of the fMRI image was reduced by sampling the BOLD signals using 560 large voxels, which are of the size 16 × 16 × 16 mm instead of the commonly used voxel size of 3 × 3 × 3 mm. Then, the BOLD signal was modeled using a general linear model to produce a *β*-value for each voxel (see **Methods **section). Using values from the voxels as input features, their in-house-developed SVM model achieved an outstanding accuracy in the study. Adopting a similar strategy, we tested the performance of SVM on the re-sampled BOLD signals from the subjects of this study, and the overall SVM classification accuracy was less than 60%. The discrepancy between our results and those from Davatzikos *et al. *can be possibly explained by the differences in the subject populations and parameterization of SVM models. While the resampling approach alleviates the difficulty of high dimensionality, large voxels (~100 times the size of conventional voxels) may potentially result in the loss of information regarding the anatomic structure involved in the cognitive process of deception. This prompted us in the direction of identifying the most discriminative features through feature selection, instead of through the dimension reduction approach.

### Classification with feature selection

Methods for feature selection are mainly grouped into two categories: the filter approach; and the wrapper approach [17,18]. In the filter approach, feature selection is only based on predefined relevance measures and is independent of classification performance of specific classifiers. We investigated five feature selection approaches, including two filter methods, Fisher criterion score (FCS) [19] and Relief-F [20], two wrapper methods, GAR2W2 and GAJH [21], and an ensemble method. Both wrapper methods use SVM as the classifier and the genetic algorithm (GA) [22] to search for the 'fittest' feature subset. The ensemble method is designed based on FCS, Relief-F, GAR2W2 and GAJH (see **Methods **section). We applied these five methods on the data with 65,166 features to identify the discriminative features.

**Figure 1:**
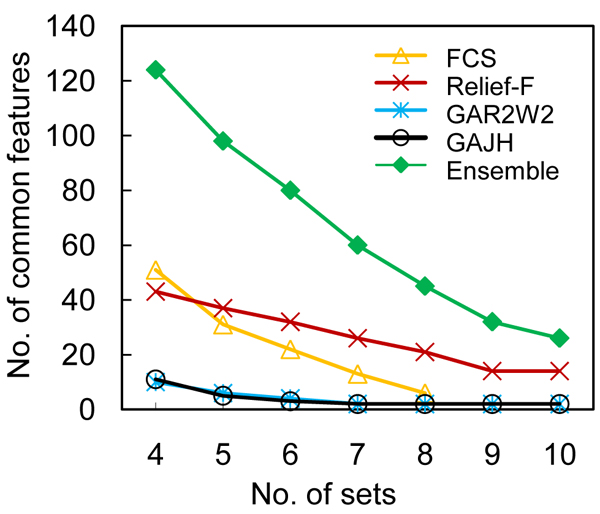
**The number of the identified common features**. The figure plots the number of the identified common features against the number of sets sharing them.

To a varying degree, selection of features is a greedy process for all above methods. As such, the results will be biased by the samples used for training, especially when the number of training cases is small. In order to address this problem, we performed a ten-fold feature selection to identify the features that consistently performed well. The data set was divided into 10 folds. During each iteration, we held one fold, put the other 9 folds of data together and selected a set of 50 features based on these 9 folds. We repeated the process 10 times, which led to 10 sets of features for each approach: FCS, Relief-F, GAR2W2 and GAJH. To evaluate the consistency of each of these four methods, we identified the features that were shared by 4 sets up to 10 sets. Furthermore, we also identified the common features from all sets derived using the above four methods, which can be referred to as an ensemble method for feature selection. The number of the identified common features is plotted against the number of sets sharing them in Figure 1. Comparing the within-methods consistencies of the two filter methods, we found that the FCS method returned no features that were share by more than 8 fold sets, while the Relief-F method returned more features shared by more sets. The results indicated that feature selection according to FCS tended to be more easily biased by training data than Relief-F. With respect to the between-methods-consistencies, the features returned by the two methods are largely disjointed: only 6 common features were shared in both sets of 51 features (FCS) and 43 features (Relief-F), which indicates that different criteria return different features. Similar analysis on the features returned by wrapper methods showed that the number of common features was relatively small for the two GA-based methods. This reflects the nature of GA in that selection is stochastic and a large number of combination of features can have a similar fitness, due to the fact that GA does not select individual features based on their ranking. Instead, it attempts to identify the best combination of features for classification. However, out of the 11 and 10 features returned by GAR2W2 and GAJH, respectively, 5 features were returned by both methods – a significant intersection. Thus, although fewer common features were identified by each of the methods, the two wrapper methods using different criteria were capable of identifying the same features that were deemed discriminative. The figure also shows that, as expected, more selected features were shared in 4–10 fold sets when the ensemble of 40 sets was pooled. There were 124 common features shared in 4 fold sets using the ensemble method, which were later mapped back to the brain volume to identify regional brain activation patterns associated with deception activities.

**Figure 2:**
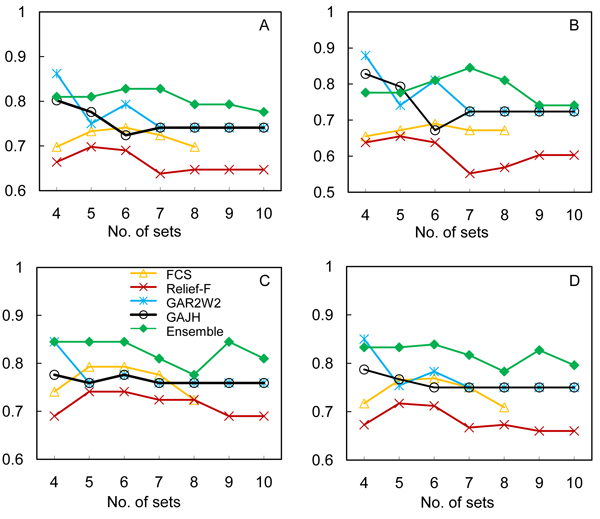
**The classification performance using the selected features**. (A) Classification accuracy. (B) Sensitivity. (C) Specificity. (D) PPV. The leave-one-out procedure was employed to evaluate the performance of SVM in deception detection using the selected features. The performance was measured in terms of accuracy, sensitivity, specificity and PPV.

To evaluate the impact of feature selection on classification performance, we used the common features identified in the above steps from each method to train SVM and evaluated the performance with the leave-one-out procedure. The classification performance was measured in terms of accuracy, sensitivity, specificity and positive predictive value (PPV), and is shown in Figure 2A–D. Although each of the filter methods identified more common features, they were not necessarily discriminative. The performance using the features returned by both FCS and Relief-F was worse than those using the wrapper methods. Although fewer shared features were returned by the wrapper methods in comparison to filter methods, these features overall performed better than those derived from the filter methods in terms of accuracy and sensitivity. The observed superiority may be attributed to the consistency of the feature selection and the relevance of the features because a classifier and its theoretical error bounds were involved in feature selection. Overall, the feature sets returned by the ensemble feature selection method outperformed those from each individual method. There are two potential advantages of the ensemble feature selection method. First, it combines the features selected according to different selection criteria, and thus covers different aspects of discrimination between classes. We noted that in Panel C of Figure 2, the sensitivity of classification for the ensemble method shows a "bell-shaped" curve, in which the set of 60 features (shared by 7 fold sets) provides the most discriminative power. From the ensemble method, the numbers of features shared by 4–10 candidate sets are greater than those from the individual feature selection methods. The additional features potentially make the classifier more resistant to variances in the signals associated with each feature. However, this does not indicate that more features result in better performance.

### Anatomic locations of the selected features

**Figure 3:**
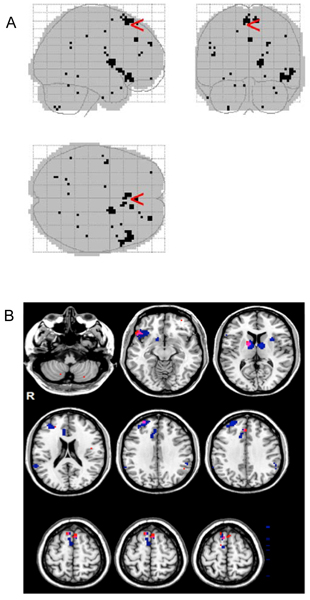
**The anatomic locations of the selection features**. (A) The 124 selected features from the ensemble method were mapped back to the brain volume. (B) Overlay of the selected features to the regions identified by group-wise statistical analysis. The 124 selected features from the ensemble method were mapped to the brain regions identified by group-wise statistical test. In the figure, the 124 selected features are marked with the red color and the regions identified by group-wise statistical test are marked with the blue color.

We mapped the 124 selected features from the ensemble method back to the brain volume and inspected the feature locations, which are shown in Figure 3. Panel A of the figure shows the localization of the voxels selected as discriminative features and Panel B shows the overlapping of the features selected in our previous and this study. Interestingly, although the features are far apart in the input feature vector for classifiers, they tend to form clusters in the brain regions (Figure 3A). The corresponding brain regions, reported for clusters of at least 2 contiguous voxels, were bilateral supplementary motor area (BA 6 and 8), right medial superior frontal cortex (BA 8 and 32), right middle frontal gyrus (BA 46), left Rolandic operculum (BA 48), right putamen and pallidum, right inferior orbitofrontal cortex (BA 11/38), and left cerebellum. Supplementary motor area (BA 6 and 8) is one of the most frequently reported areas across studies of deception [23]. There is general correspondence (Figure 3B) between brain regions identified in the present analysis and brain regions previously reported to have predictive value in detecting deception, such as supplementary motor areas (BA 6 and 8), right inferior orbitofrontal cortex (BA 38), and right middle frontal gyrus (BA 46) [5].

The anatomic overlapping of the selected features with the brain regions from the previous studies indicates that the feature selection procedure provides another means to identify the brain regions that may be involved in the psychological process of deception. However, it should be noted the method in this study for identifying the brain regions is significantly different from the previous ones. In the previous analysis [5], all fMRI images associated with one type of event, e.g., *truth *or *lie*, were pooled from all subjects, followed by group-wise comparison to identify the regions with differential brain activities. Therefore, the procedure was not classification oriented, and the features from this procedure resulted in a relatively poor classification performance using our methods. On the other hand, the feature selection approach from this study combines the procedure of using t-maps to remove background noise and selecting features that are relevant to the classification. Therefore, it is not surprising that the features from this task-oriented procedure significantly enhanced classification performance.

## Conclusion

In this study, we investigated the utility of feature selection in fMRI-based detection of deception. The high dimensionality of fMRI data makes it a difficult task to directly utilize the original data as input for classification algorithms. Our results indicate that feature selection not only enhances classification accuracy in fMRI-based detection of deception when compared to the models that rely on dimension reduction, but also provides support for the biological hypothesis that brain activities in certain regions of brain are important for the discrimination of deception. While these conclusions were obtained in the setting of detection of deception, the general approach of feature selection is applicable to the identification of other fMRI-based biomarkers.

## Methods

### fMRI data processing

The data are from a previously published study of fMRI detection of deception [5]. Briefly, a test participant was instructed to take a ring or a watch before the fMRI scan and was asked to lie regarding which object he/she took. Then, fMRI scans were acquired while the subject responded to the visually presented questions. Four types of questions were asked: neutral, truth, lie and control. All images associated with one type of event from a subject were grouped and were modeled with a general linear model using the statistical parametric mapping (SPM2) software package [24]. For each subject, the procedure produced 4 parametric maps, referred to as *β*-maps, corresponding to the 4 types of questions. Each voxel in these maps contained the estimated parameter of the general linear model, reflecting the influence of the event on the BOLD signal. Pooling the data from 61 subjects led to a data set consisting of 61 *β*-maps labeled with *truth *class and 61 maps labeled as *lie *class. In order to normalize the influence of the events, the *β*-maps corresponding to the lie and truth events were further compared to that of the neutral event to produce truth-vs-neutral and lie-vs-neutral *t*-statistics maps, referred to as t-maps. A standard gray-matter map was applied to the *β*-maps and t-maps, such that the values of 65,166 voxels corresponding to brain gray matter were retained and used as input features for classification. Through a series of test, we found that the classification performances of all classifiers were consistently better using t-maps rather than *β*-maps (data not shown) as input. Therefore the following results reflect using t-maps as input features.

### Classification without feature selection

In order to make a comparison to classification with feature selection in deception detection, partial least square (PLS) [11], random forest (RF) [12] and support vector machine (SVM) were used to perform high-dimensional classification without feature selection. We have used LIBSVM [25], a library for SVM and packages of PLS and RF implemented in the R language downloaded from the Comprehensive R Archive Network (CRAN) [26].

### Feature selection methods

We employed the Fisher criterion score (FCS) and Relief-F, which are two filter methods for feature selection. FCS ranks features according to their capability to separate different classes [19] and ignores feature dependence. For a problem with two classes, a feature's FCS is calculated (as Equation (1)) by dividing the distance between the feature's means by the sum of variance within each class. In Equation (1),  and  are means of feature *i*'s values belonging to the positive and negative classes respectively, and  and  are the feature *i*'s standard deviations of the positive class and the negative class, respectively.

(1)

On the other hand, Relief-F [20] uses a weighting approach to rank features. For a two-class task, Relief-F repeatedly draws a random instance from the data set. Then, *k *nearest neighbors of the instance are selected from the same class and the opposite class, leading to two sets of cases. Iterating through each feature, the weight associated with the feature is adjusted. The weighting scheme strives to minimize the averaged distance, evaluated with a feature of interest, of the instance to its neighbors of the same class while maximizing the averaged distance to the neighbors of the different class.

**Figure 4:**
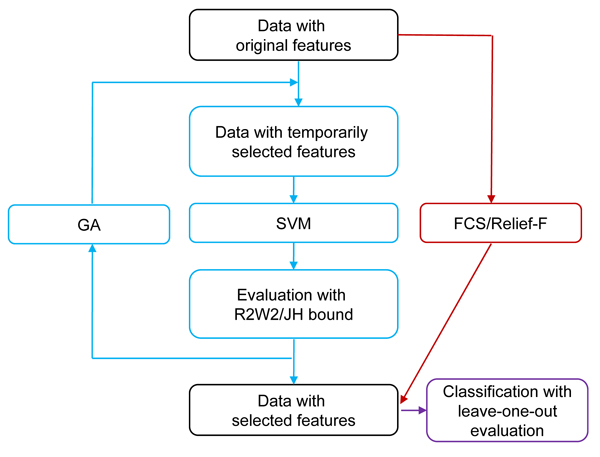
**Procedure of feature selection with the filter and wrapper methods**. In the figure, GAR2W2 and GAJH follow the procedure marked with the blue color; FCS and Relief-F flow the dark-red-colored path.

We also employed two wrapper methods proposed and implemented by Fröhlich *et al *[21] for feature selection. Both wrapper methods use SVM as the classifier and the genetic algorithm (GA) [22] to search the feature space for the 'fittest' feature subset. GA [22] is an optimization algorithm inspired by biological principles of evolution. In GA, features are encoded as alleles on a chromosome, in which a "1" indicates the corresponding feature (allele) is selected for classification. A fittest feature set is identified by a series of random mutations and crossovers of chromosomes. During iterations, the features were selected by the GA algorithm, and the performance criteria of the SVM based on the selected features were used as fitness functions for the GA algorithm for further selection. In this study, two criteria – the radius margin (R2W2) bound [27] and the Jaakkola-Haussler (JH) bound [27-30] – were used as fitness functions for GA algorithms. These criteria reflect the theoretical leave-one-out error bounds of an SVM based on a given set of features. The wrapper feature selection methods based on these criteria are referred to as GAR2W2 and GAJH [21], respectively. The overall procedure of feature selection with FCS, Relief-F, GAR2W2 and GAJH is shown in Figure 4.

For a nonlinear problem, SVM tries to find an optimal soft margin hyperplane by solving the following dual problem

(2)

where  and  are training samples, *C *is the regularization parameter used to trade-off between margin maximization and error minimization, *K *is a kernel function, the size of the training data set is *n*, and *α *= (*α*_1_, ⋯, *α*_*n*_).

As defined in Equation (3), the R2W2 bound for the expectation of the test error is defined based on the square of the ratio of the radius of the sphere enclosing the training data over the margin of the hyperplane separating the data.

(3)

where α* is the solution of Equation (2), and 1/*W*^2^(α*) is the size of the maximal margin. The radius *R *of the sphere is calculated as follows,

(4)

where *β *= (*β*_1_, ⋯, *β*_*n*_).

The JH bound is defined as Equation (5) for an SVM without threshold. The function *ψ *(*v*) in the equation is equal to 0 if *v *≤ 0; 1 otherwise.  is the probability of test error of an SVM classifier built on the training set of size *n*-1.

(5)

## Competing interests

Steven Laken, Ph.D. holds stock in Cephos Corporation and is an officer of the company. Further, he receives compensation from Cephos and may benefit from stock and stock options in Cephos Corporation. The Medical University of South Carolina Foundation for Research Development holds stock in Cephos Corporation.

## Authors' contributions

XL conceived and directed the project; BJ carried out the computational experiments; AS produced the images of Figure 3; SJL, FAK, KAJ, and MSG conducted the fMRI and deception experiments. XL and BJ drafted the manuscript and all authors contributed to the editing of the manuscript.
